# Testing telediagnostic right upper quadrant abdominal ultrasound in Peru: A new horizon in expanding access to imaging in rural and underserved areas

**DOI:** 10.1371/journal.pone.0255919

**Published:** 2021-08-11

**Authors:** Thomas J. Marini, Daniel C. Oppenheimer, Timothy M. Baran, Deborah J. Rubens, Ann Dozier, Brian Garra, Miguel S. Egoavil, Rosemary A. Quinn, Jonah Kan, Rafael L. Ortega, Yu T. Zhao, Lorena Tamayo, Claudia Carlotto, Benjamin Castaneda

**Affiliations:** 1 Department of Imaging Sciences, University of Rochester Medical Center, Rochester, New York, United States of America; 2 Department of Public Health, University of Rochester Medical Center, Rochester, New York, United States of America; 3 Medical Imaging Ministries of the Americas, Clermont, Florida, United States of America; 4 Medical Innovation and Technology, San Isidro, Lima, Peru; 5 Department of Engineering, Pontifica Universidad Catolica del Peru, San Miguel, Lima, Peru; University of Oklahoma Health Sciences Center, UNITED STATES

## Abstract

**Background:**

Hepatic and biliary diseases are prevalent worldwide, but the majority of people lack access to diagnostic medical imaging for their assessment. The liver and gallbladder are readily amenable to sonographic examination, and ultrasound is a portable, cost-effective imaging modality suitable for use in rural and underserved areas. However, the deployment of ultrasound in these settings is limited by the lack of experienced sonographers to perform the exam. In this study, we tested an asynchronous telediagnostic system for right upper quadrant abdominal ultrasound examination operated by individuals without prior ultrasound experience to facilitate deployment of ultrasound to rural and underserved areas.

**Methods:**

The teleultrasound system utilized in this study employs volume sweep imaging and a telemedicine app installed on a tablet which connects to an ultrasound machine. Volume sweep imaging is an ultrasound technique in which an individual scans the target region utilizing preset ultrasound sweeps demarcated by easily recognized external body landmarks. The sweeps are saved as video clips for later interpretation by an experienced radiologist. Teleultrasound scans from a Peruvian clinic obtained by individuals without prior ultrasound experience were sent to the United States for remote interpretation and quality assessment. Standard of care comparison was made to a same-day ultrasound examination performed by a radiologist.

**Results:**

Individuals without prior ultrasound experience scanned 144 subjects. Image quality was rated “poor” on 36.8% of exams, “acceptable” on 38.9% of exams, and “excellent” on 24.3% of exams. Among telemedicine exams of “acceptable” or “excellent” image quality (n = 91), greater than 80% of the liver and gallbladder were visualized in the majority of cases. In this group, there was 95% agreement between standard of care and teleultrasound on whether an exam was normal or abnormal, with a Cohen’s kappa of 0.84 (95% CI 0.7–0.98, p <0.0001). Finally, among these teleultrasound exams of “acceptable” or “excellent” image quality, the sensitivity for cholelithiasis was 93% (95% CI 68.1%-99.8%), and the specificity was 97% (95% CI 89.5%-99.6%).

**Conclusion:**

This asynchronous telediagnostic system allows individuals without prior ultrasound experience to effectively scan the liver, gallbladder, and right kidney with a high degree of agreement with standard of care ultrasound. This system can be deployed to improve access to diagnostic imaging in low-resource areas.

## Introduction

Neoplastic and non-neoplastic forms of liver disease are increasing in prevalence around the world and have significant implications for the health of the global community [1–3]. Biliary disease including cholelithiasis is another global cause of morbidity and mortality [4–7]. Gallstones occur in up to 20% of the population worldwide, and the incidence is rising secondary to increasing obesity [4, 6–8]. Although hepatic and biliary diseases are frequently encountered, the majority of people worldwide lack access to diagnostic medical imaging for their assessment [9–11]. The liver and gallbladder are readily amenable to sonographic examination which may assist in the diagnosis of pathology and alter patient management [12]. Detecting such pathology by ultrasound is particularly important in resource-poor countries which may not have more advanced imaging techniques available, such as computed tomography or magnetic resonance imaging [10, 11].

While ultrasound is a portable, safe, and cost-effective imaging modality, it traditionally requires a highly experienced operator to obtain diagnostic-quality images. Furthermore, if a practitioner is not available to interpret the ultrasound at the point of care, the images must then be transferred to a radiologist for interpretation, but the telehealth infrastructure in underserved areas is often limited or non-existent [13–15]. A telediagnostic system, previously piloted in Peru, has several key features which circumvent these issues (S1 Table), including the ability to transmit diagnostic images over low bandwidth and obtain imaging in the absence of an on-site specialist [15]. In this telediagnostic system, imaging acquisitions are acquired by operators trained on volume sweep imaging (VSI) ultrasound scan protocols based on easily identified external body landmarks. The VSI ultrasound protocols require no significant anatomic knowledge or complex technical skill to perform. Imaging acquisitions are saved as cine clips, which are sent via a telemedicine platform for remote asynchronous interpretation by the diagnostic radiologist.

**Fig 1:**
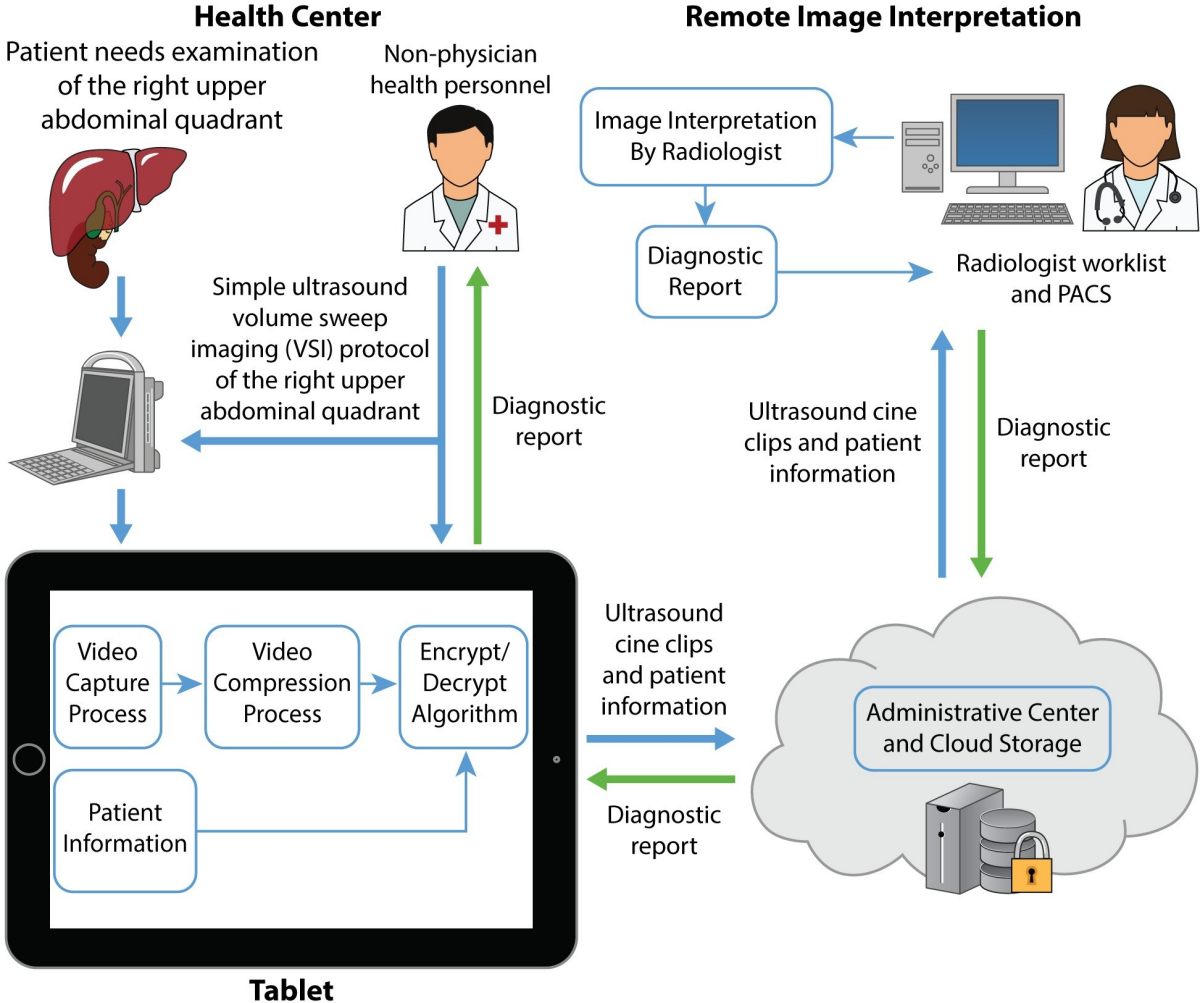
Overview of the RUQ telediagnostic ultrasound system. Imaging information of the right upper abdominal quadrant is acquired by health personnel of any skill level and is sent through the tablet from the health center for remote imaging interpretation (blue arrows). A diagnostic report is returned to the health center for the health personnel and patient (green arrows).

Many studies have shown the promise of teleultrasound in improving global health, but none directly focus on the right upper abdominal quadrant [13]. Furthermore, these studies often involve real-time synchronous videoconference systems that rely on high bandwidth and the concurrent availability of a specialist. The asynchronous teleultrasound system used in this study requires neither. Previous pilot data of the telediagnostic system used in this study show significant promise for right upper quadrant (RUQ) abdominal scanning indications [15]. In that study, the telediagnostic approach visualized the liver, gallbladder, and right kidney with acceptable or excellent image quality in 85% of cases. To address limitations of the pilot study, including low sample size and lack of a reference standard for comparison, in this study, we compared the telediagnostic system for RUQ abdominal ultrasound scanning with standard of care RUQ ultrasound performed by a radiologist in a Peruvian clinic. The telediagnostic system for RUQ ultrasound is shown in Fig 1. Based on our previous experience, we hypothesized that the interpretations from the telediagnostic system’s imaging would be concordant with those from standard of care RUQ ultrasound. The goal of this study was to provide a concrete proof of concept for RUQ teleultrasound.

## Materials and methods

### Right upper abdominal quadrant VSI

VSI was developed as a means to improve access to ultrasound imaging in underserved areas. In this imaging technique, an operator sweeps the ultrasound probe over the target region to obtain cine clips. The start and stop points of the sweeps are all based on easily recognized external body landmarks. The ideal probe speed is 1–2 cm/s, producing an image every 1–2 mm. Probe angulation at the beginning and end of the sweep maximizes the area of interest imaged. The cine clips of a single protocol consist of a full volumetric acquisition of the target region, which are then sent for remote interpretation by an expert. The individual performing the protocol is instructed not to look at the ultrasound screen but to focus on the probe position relative to the patient’s external landmarks. The VSI operator does not perform any image interpretation. A general abdominal preset eliminates the need for technical adjustments of transducer frequency, gain, or focal zone.

Previous detailed study of a lung VSI protocol showed that within one hour, individuals could perform probe sweeps with basic competence [16]. In our experience, 8 hours is sufficient to teach and reinforce a RUQ VSI protocol, including training on the telediagnostic system [15]. Individuals without prior imaging experience or medical training can perform VSI effectively; the only true requirements involve the ability to hold the probe and follow simple instructions [15–18]. In general, a VSI examination with the RUQ protocol is usually performed within 10 minutes or less. Interpretations can similarly be performed in a 10-minute time frame.

**Fig 2:**
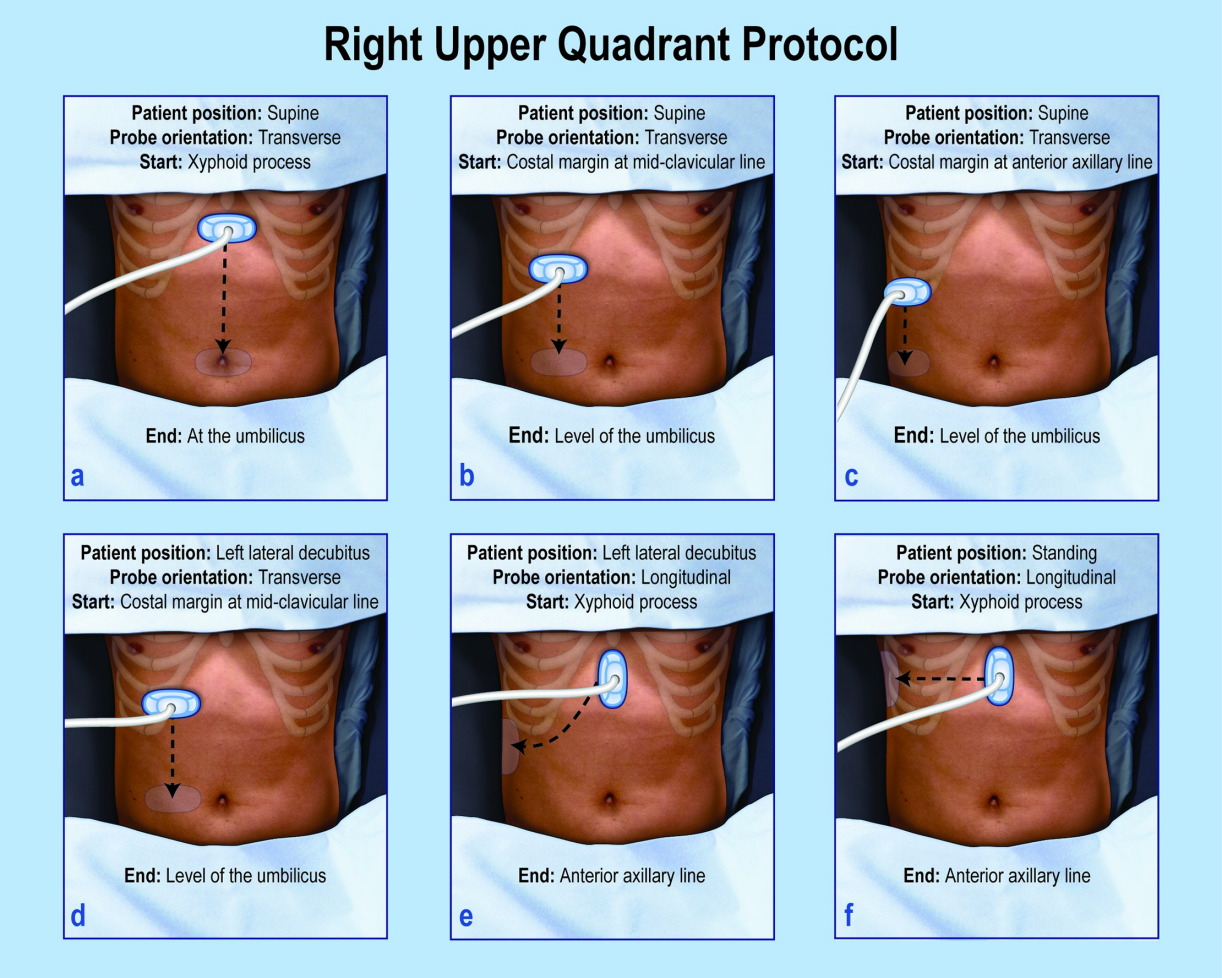
Overview of the RUQ protocol. The RUQ protocol involves 6 sweeps each beginning and ending with an arc of the probe that has not been illustrated for simplicity. An arc consists of a fan-like motion of the probe (shown in S1 File). The arcing of the probe allows maximal visualization. Steps a-c involve transverse sweeps of the probe in the supine position. Steps d and e are conducted in the left lateral decubitus position. Step f is performed in the standing position. The patient takes a deep breath during each step.

The abdominal VSI protocol provides a comprehensive examination of the abdominal RUQ in Fig 2. The patient is instructed to fast for 6 hours prior to the exam and to take a deep breath and hold it for each VSI sweep. The patient changes position during the exam to assess for gallstone mobility. All images in this study (standard of care and VSI) were obtained with a portable Mindray DP-10 ultrasound machine (Mindray, China) using a 4.5 Hz curvilinear probe and an abdominal preset. A video on how to perform the VSI protocol and a poster of the complete VSI protocol used to train the operators are available as supporting material (S1 File).

### Telemedicine system

The telemedicine system used in this study is called “Medical for Ultrasound” or MED4US (Medical Innovation and Technology, Peru). The technical specifications of MED4US have been previously described, and it has been shown to send imaging acquisitions over low Internet speeds (150 kbps) in reasonable send times [19]. MED4US is installed on a standard Windows 10 tablet which connects to the ultrasound machine via a video to USB converter. The system was designed to be user-friendly and upon opening, guides the operator into choosing the type of VSI exam and entering relevant clinical data. Once the clinical information is entered, the program guides the operator in performing each step of the VSI protocol. Upon completing each cine clip in the protocol, the program compresses, encrypts, and uploads the imaging acquisition to a secure server. Because this is an asynchronous system, imaging clips can be uploaded any time after acquisition, meaning clips can be obtained even in the absence of an Internet connection. Once uploaded, the images are available for a remote radiologist to review and produce a diagnostic report that can be returned to the patient and health center.

### Study design

This study was approved by the Institutional Review Board at the Hospital Nacional Docente Madre Niño San Bartolome. The RUQ telediagnostic ultrasound was tested in the Conde de la Vega Health Center in Lima, Peru. The center serves a low-income and underserved area of Lima and has a busy practice environment. A low to moderate speed 3G Internet connection is available at the center.

A 32-year-old care technician and a 42-year-old nurse without prior ultrasound experience underwent training on the VSI protocol and the telemedicine system in May 2018. Training took about 8 hours and involved didactic and hands-on training. At the end of the session, both operators were directly supervised by the study team and noted to perform the protocol accurately. Official study data collection began in June 2018 and ended in March 2019. Individuals older than 18 years of age visiting the clinic were offered enrollment in the study in a convenience sampling scheme at the availability of the Peruvian radiologist, who would perform the standard of care imaging. Inclusion criteria included attendance at the clinic and fasting for at least 6 hours prior to the ultrasound exam. None of the subjects scanned had acute gastrointestinal symptoms. Those enrolled were presenting for routine follow-up appointments not related to gastrointestinal disease. At the time of the appointment, the radiologist informed the patient of the study and inquired as to the time of his or her last meal. If the patient expressed interest in the study and had not eaten within 6 hours, the radiologist proceeded to obtain informed consent and enroll the patient in the study. In Peru, women seek care at clinic disproportionately to males and, in particular, this clinic specializes in obstetric and gynecological care resulting in a majority female sample [20].

Following informed consent, a standard of care ultrasound examination was performed by a Peruvian radiologist with 10 years of experience. This examination involved direct and focused examination of the liver, gallbladder, right kidney, pancreas, and large vessels in accordance with standard practice guidelines [12]. Subsequently, one of the two trained operators performed the VSI protocol and sent the images to a cloud-based server for storage and later retrieval for interpretation in the United States. Once study enrollment began, the operators who performed the VSI exams received no feedback in regards to image quality. The Peruvian radiologist had no involvement in the interpretation or acquisition of the VSI examinations.

### Remote readings

Two separate board-certified abdominal fellowship-trained American radiologists interpreted the RUQ VSI exams. They reported on the visualization of each organ (less than 30% visualization, 30–80% visualization, or greater than 80% visualization) and abnormalities of the liver, gallbladder, pancreas, and right kidney. Liver echogenicity was considered separately from whether the liver or overall exam interpretation was rated as abnormal to simplify analysis. To prevent arbitrary disagreement among hepatic echogenicity categories (i.e., normal liver echogenicity versus mildly increased echogenicity/hepatic steatosis), hepatic echogenicity was scored as normal/mildly increased echogenicity or moderately/severely increased echogenicity (hepatic steatosis). For each category, the VSI readers also recorded the confidence in their assessments (“confident,” “intermediate confidence,” or “not confident”). The overall image quality was rated as “excellent,” “acceptable,” or “poor.” “Excellent” examinations showed complete or nearly complete visualization of the liver and gallbladder with appropriate imaging quality. “Acceptable” examinations showed nearly complete or partial visualization of the liver and gallbladder with appropriate imaging quality. “Poor” examinations showed only partial or inadequate visualization of the liver and gallbladder with image quality limiting evaluation. The two radiologists described any diagnostic findings or quality issues in additional free text comments.

### Statistical analysis

Abnormalities and visualization were summarized across readers by rate of occurrence and 95% confidence interval (CI). Agreement between VSI and standard of care ultrasound was quantified using Cohen’s kappa. These kappa values were then compared to a theoretical mean of 0 using one-sample t-tests. Differences in file size and sweep length were compared based on image quality using ordinary one-way ANOVA. Confidence was summarized by the median rating and range and was compared between groups using the chi-square test. In order to examine whether VSI operators changed image quality over the study, multinomial regression with image quality as the outcome and date and time of scan as predictors was performed. Further linear regression was performed with sweep length as the outcome and date and time of scan as predictors in order to determine whether operators changed sweep length over the course of study. In both cases, date was coded as the number of days from the first exam recorded in the study, and time was coded as the number of hours from midnight. Analysis and results for examinations with “excellent” or “acceptable” image quality (n = 91) are the primary focus for analysis with full results including examinations of “poor” quality reported in supplemental material. All statistical analysis was performed using SPSS (v26, IBM Corporation, Armonk, NY) and MATLAB (R2019b, MathWorks, Natick, MA).

## Results

144 subjects were scanned (129 female and 15 male) with an average age of 43.9 years (age range 18–90 years). Demographic breakdown and image quality is shown in S2 Table. No significant difference between male and female subjects was identified (p = 0.57). There were 25 abnormal scans, most attributed to gallstones (n = 19) and only a few cases of liver/kidney cysts (n = 5). The remaining abnormal case represented a gallbladder polyp. Hepatic steatosis was considered in a separate analysis for simplification, and there were 25 cases in the study sample. There were no significant differences in sweep length or file size between “acceptable”/ “excellent” quality exams compared with “poor” quality exams (S3 Table). Image quality and sweep length did not vary by either date or time of scan, indicating these metrics remained stable over the course of study (S4 Table). As expected, exams of higher image quality resulted in higher levels of confidence in the findings (S5 Table).

Examination image quality and visualization of each organ for all examinations is shown in Table 1. Among all exams, 36.8% were rated of “poor” image quality, 38.9% of “acceptable” image quality, and 24.3% of “excellent” image quality. When considering only the “acceptable” and “excellent” exams, 61.5% were “acceptable” and 38.5% were “excellent.” The liver and gallbladder showed greater than 80% visualization in a majority of the “acceptable” and “excellent” image quality cases. Agreement was calculated between VSI and standard of care and is shown in Table 2 for examinations of at least “acceptable” image quality. S6 Table shows agreement for all examinations. Overall agreement was high among examinations of at least “acceptable” quality, with Cohen’s kappa indicating good to excellent agreement between VSI and standard of care ultrasound for all but right kidney abnormality. When VSI cases that did not visualize the organ in question were excluded, overall agreement increased, and Cohen’s kappa indicated good to excellent agreement for all abnormalities. Finally, the sensitivity and specificity for cholelithiasis was calculated (Table 3). 93.3% (68.1–99.8%) sensitivity and 97.0% (89.5–99.6%) specificity were achieved for cholelithiasis among exams of “acceptable”/“excellent” image quality.

**Table 1:** Image quality and organ visualization of all examinations.

Measure	Level	Percentage (95% confidence interval)	
		All Exams	Acceptable/Excellent Image Quality
Image Quality	Poor	36.8% (28.9–45.2%)	-
	Acceptable	38.9% (30.9–47.4%)	61.5% (50.8–71.6%)
	Excellent	24.3% (17.6–32.1%)	38.5% (28.4–49.2%)
Gallbladder Visualization	<30%	23.6% (16.9–31.4%)	7.69% (3.15–15.2%)
	30–80%	22.2% (15.7–29.9%)	14.3% (7.83–23.2%)
	> = 80%	54.2% (45.7–62.5%)	78% (68.1–86%)
Pancreas Visualization	No	56.3% (47.7–64.5%)	46.2% (35.6–56.9%)
	Partial/complete	43.8% (35.5–52.3%)	53.8% (43.1–64.4%)
Right Kidney Visualization	<30%	35.4% (27.6–43.8%)	11% (5.4–19.3%)
	30–80%	36.1% (28.3–44.5%)	44% (33.6–54.8%)
	> = 80%	28.5% (21.3–36.6%)	45.1% (34.6–55.8%)
Liver Visualization	<30%	19.4% (13.3–26.9%)	2.2% (0.267–7.71%)
	30–80%	38.9% (30.9–47.4%)	33% (23.5–43.6%)
	> = 80%	41.7% (33.5–50.2%)	64.8% (54.1–74.6%)

**Table 2:** Agreement between VSI and standard of care for acceptable/excellent image quality.

Measure	VSI	Standard of Care Ultrasound	Overall Agreement	Overall agreement (ignoring non-visualized cases)	Cohen’s kappa (95% confidence interval)	Cohen’s kappa (ignoring non-visualized cases)	P value	P value (ignoring non-visualized cases)
Liver Echogenicity (% Normal)	86.8% (78.1–93%)	86.8% (78.1–93%)	100%	100%	1(1–1)	1(1–1)	<0.0001	<0.0001
Liver Abnormal	2.2% (0.3–7.7%)	3.3% (0.7–9.3%)	98.9%	98.9%	0.8(0.41–1.2)	0.8(0.41–1.2)	<0.0001	<0.0001
Gallbladder Abnormal	19.8% (12.2–29.4%)	17.6% (10.4–27%)	86.8%	92.9%	0.69(0.55–0.83)	**0.8(0.65–0.95)**	<0.0001	<0.0001
Pancreas Abnormal	0% (0–4.0%)	0% (0–4.0%)	100%	100%	1*	1*	<0.0001*	<0.0001*
Right Kidney Abnormal	2.2% (0.3–7.7%)	1.1% (0.03–6.0%)	86.2%	98.7%	0.13(-0.11–0.37)	0.66(0.033–1.3)	<0.0001	<0.0001
Exam Abnormal	22% (14–31.9%)	20.9% (13.1–30.7%)	94.5%	94.5%	0.84(0.7–0.98)	0.84(0.7–0.98)	<0.0001	<0.0001

Significant agreement was found between VSI and standard of care ultrasound. When excluding cases in which a particular organ was not visualized on VSI, this agreement increased. Reported values are percentage (95% confidence interval), with Cohen’s kappa (95% confidence interval) for reader agreement. P values are results of comparing kappa to a theoretical mean of 0.

* Due to one or both sites listing all as one category.

**Table 3:** Sensitivity/Specificity for cholelithiasis.

Measure	Sensitivity (95% CI)	Specificity (95% CI)
Cholelithiasis (Acceptable/Excellent Image Quality Exams)	93.3% (68.1–99.8%)	97.0% (89.5–99.6%)
Cholelithiasis After Consensus Read (Acceptable/Excellent Image Quality Exams)	100% (78.2–100%)	97.0% (89.5–99.6%)
Cholelithiasis (All Exams)	84.2% (60.4–96.6%)	97.7% (91.9–99.7%)
Cholelithiasis After Consensus Read (All Exams)	89.5% (66.9–98.7%)	97.7% (91.9–99.7%)

Consensus reads were performed on discrepant interpretations between VSI and standard of care to assess for underlying cause. Among all examinations, there were only 9 studies with disagreement on normal versus abnormal exam and 2 studies with disagreement for liver echogenicity assessment. Upon consensus readings, 1 VSI case changed from normal to abnormal for a gallbladder finding, and 1 VSI case changed from abnormal to normal for a gallbladder finding. These were both secondary to reader error due to poor visualization of the target organ. Of note, there was a consensus read on a VSI case identifying gallstones that were missed on standard of care exam (likely due to poor visualization of the gallbladder neck on standard of care imaging). Finally, VSI detected one renal cyst missed by standard of care on consensus read. Otherwise, when VSI did not agree with standard of care on consensus read, it was almost always related to poor visualization of the target on VSI.

## Discussion

**Fig 3:**
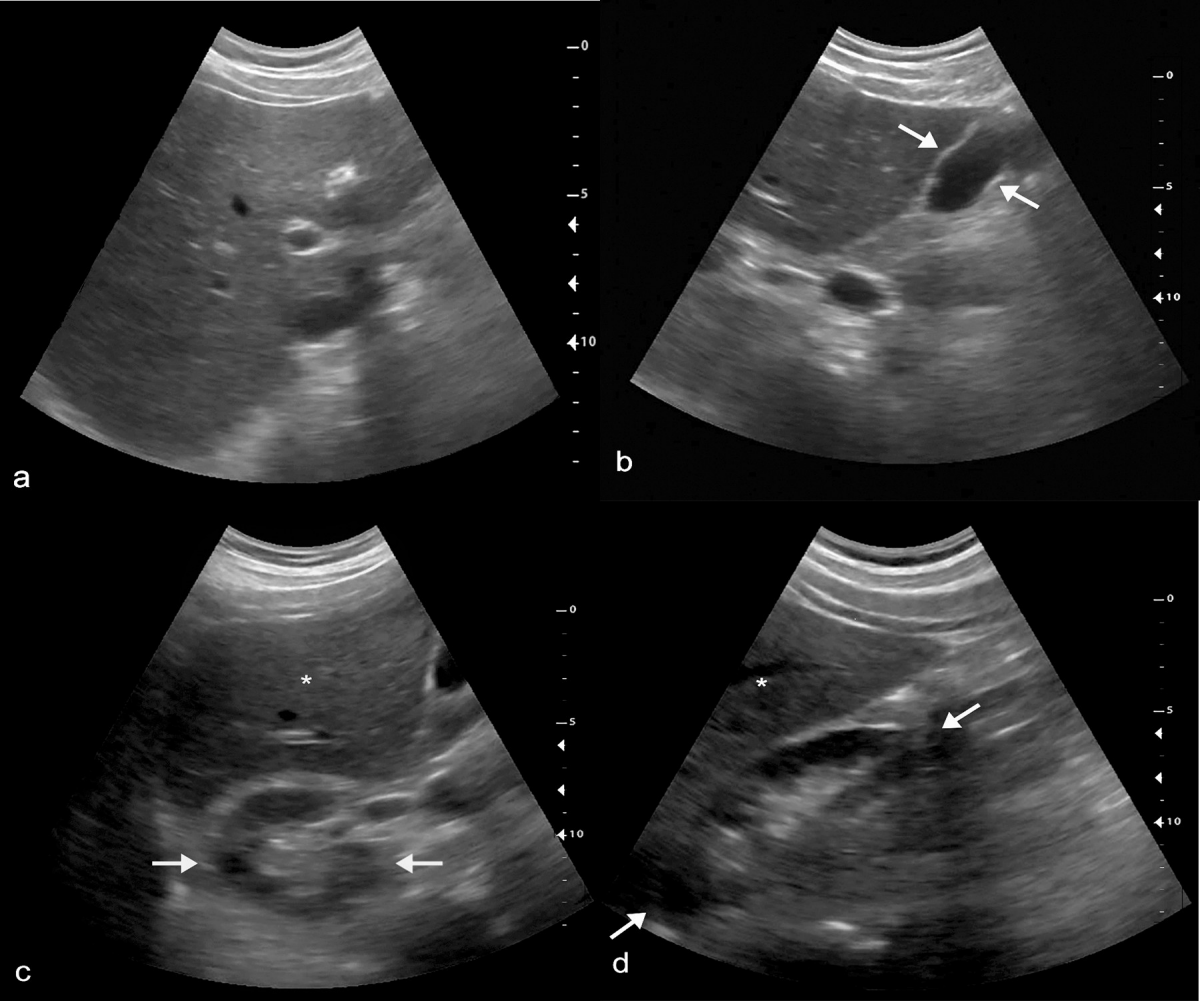
Normal RUQ VSI exam. (a) Normal liver identified on transverse VSI scan. (b) Normal gallbladder (arrows) identified on sagittal VSI scan. Transverse (c) and sagittal (d) images of the kidney (arrows) with partial visualization of the liver (*). S2 File includes the cine clips of this exam.

In this study, we tested the deployment of a telediagnostic ultrasound system for RUQ VSI in a busy health center in Lima, Peru. All imaging acquisitions were obtained by individuals without prior experience in ultrasound who received only 8 hours of training. In spite of the brief training and lack of any prior ultrasound experience, these operators produced images of sufficient diagnostic quality in the majority of cases. An example normal VSI exam is shown in Fig 3 along with supplemental material showing each sweep in the exam (S2 File). Each examination took only about 10 minutes to complete. For the relatively modest price of a tablet, a low-end portable ultrasound machine, and low-speed Internet connection, this approach could be replicated in any health clinic or community around the world.

**Fig 4:**
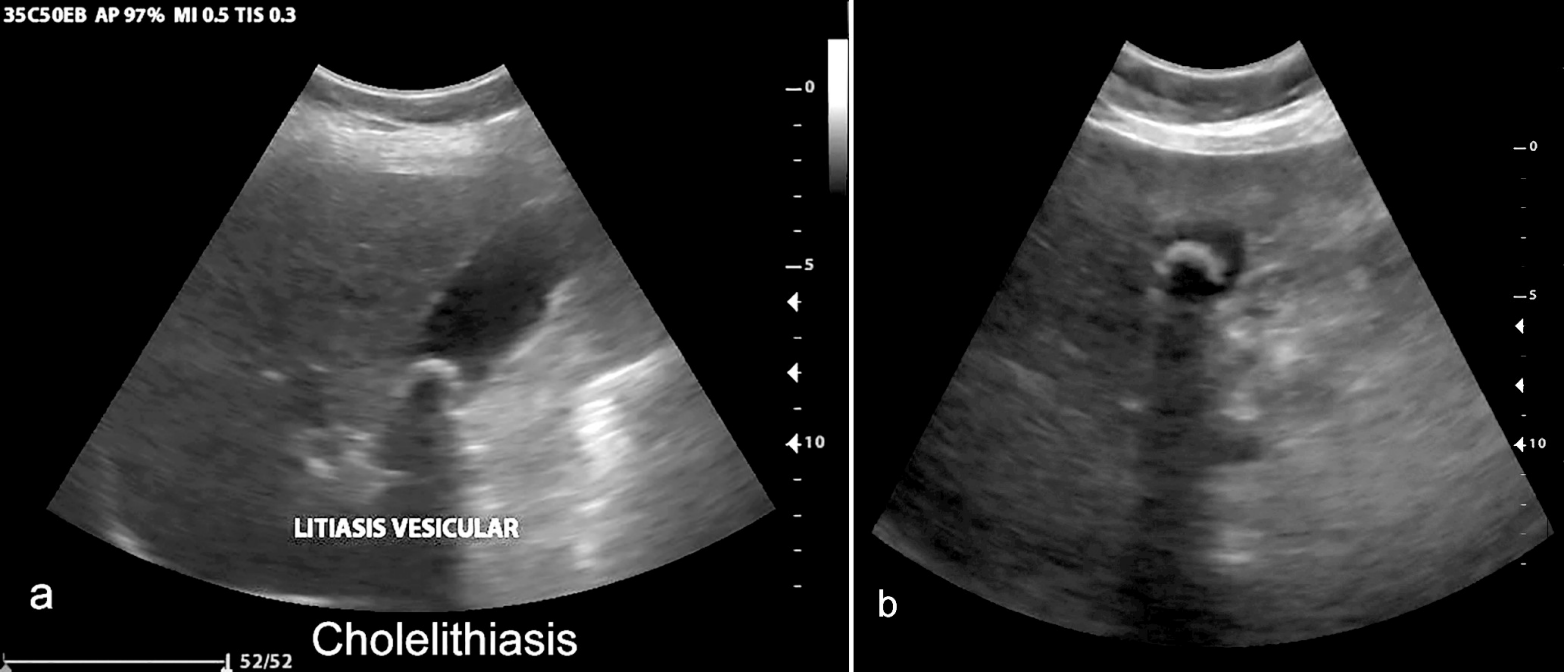
Cholelithiasis. Cholelithiasis with echogenic stone and posterior acoustic shadowing seen on standard of care (a) and VSI (b). Online Video 3 includes a sample cine clip of this study.

**Fig 5:**
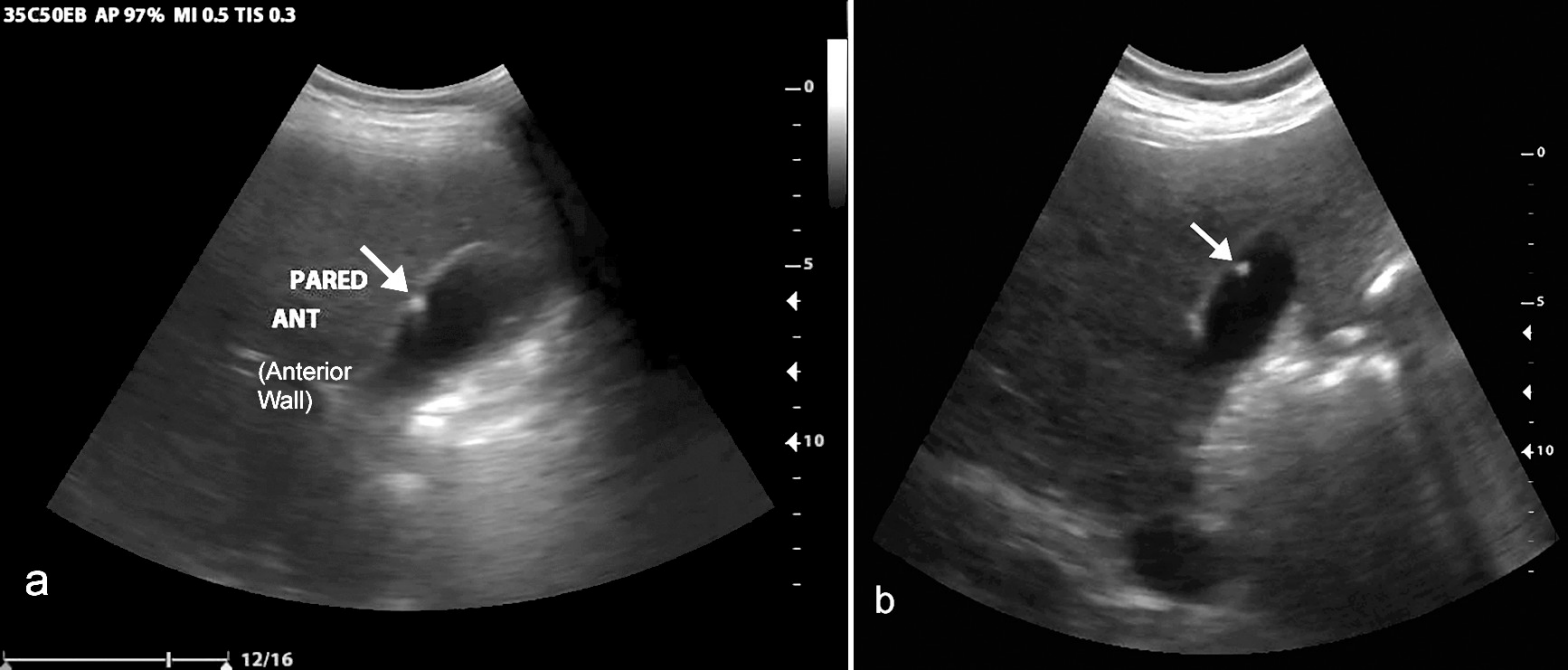
Gallbladder polyp. Gallbladder polyp with faint comet tail artifact seen on standard of care (a) and VSI (b).

Overall, there was excellent agreement between VSI performed by operators without prior training among all examinations, with approximately 95% agreement on abnormal exams. The liver, gallbladder, and kidney were at least partially visualized in the majority of examinations. Imaging of the gallbladder showed excellent sensitivity and specificity for gallstones among all exams (n = 19 gallstones were in the study sample). A case of gallstones is shown in Fig 4 and S3 File. In addition, there was one case of a gallbladder polyp shown in Fig 5, illustrating that the protocol’s utility is not just limited to cholelithiasis. Given these findings, VSI of the RUQ would also likely be effective in diagnosing acute cholecystitis, although none of the patients in this study sample had findings to suggest acute cholecystitis.

**Fig 6:**
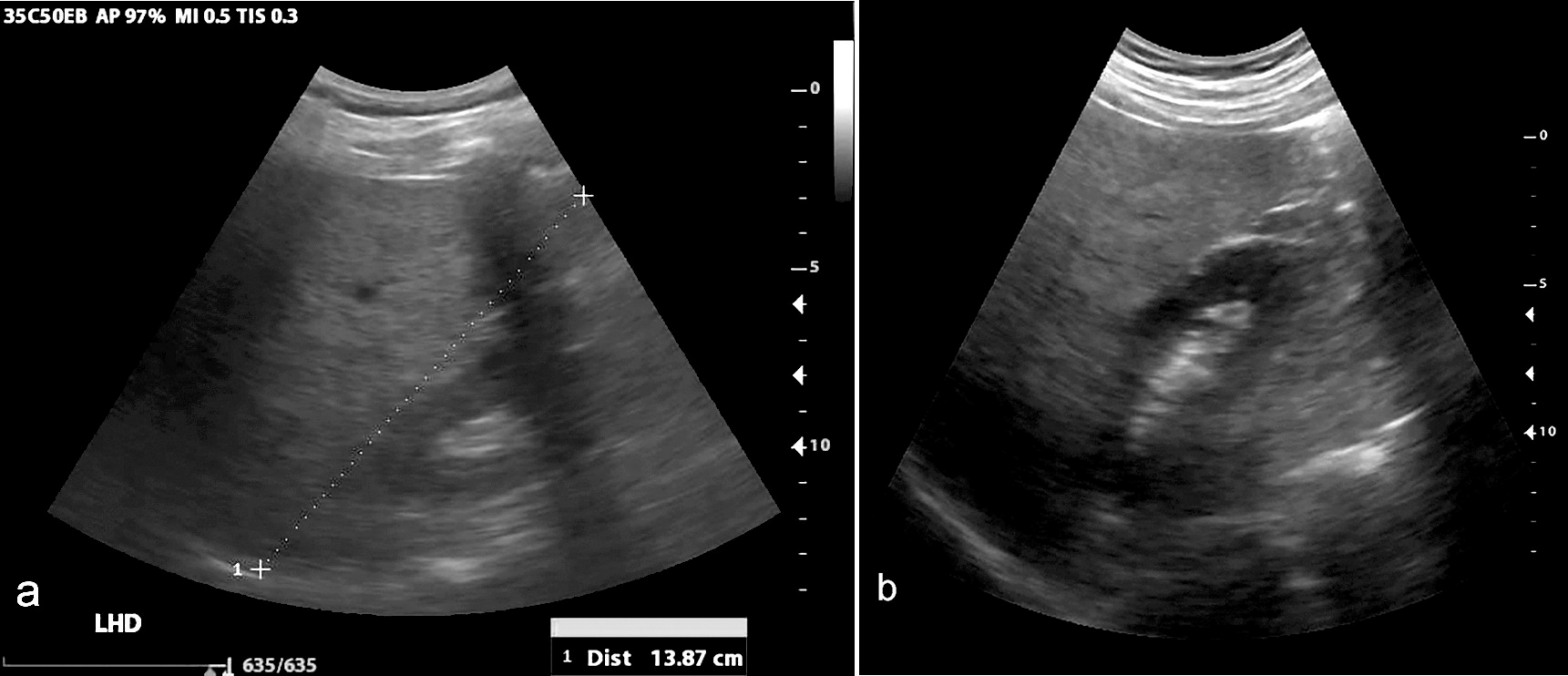
Diffuse hepatic steatosis. Diffuse hepatic steatosis seen on standard of care imaging (a) and VSI (b). In both images there is significant increase in hepatic echogenicity.

There were few focal liver lesions in our study but given that the liver was generally well visualized when the examination was of “acceptable” or “excellent” imaging quality, it stands to reason that VSI may be an effective means to evaluate for liver lesions. There was excellent assessment of hepatic steatosis, especially in examinations of “acceptable” and “excellent” imaging quality. Fig 6 shows an example of hepatic steatosis. Other structures in the RUQ like the pancreas and great vessels (along with the right kidney) are also included within the VSI sweeps, providing at least a limited examination of these structures.

While these data demonstrate the feasibility and limitations of this approach for evaluation of the RUQ, many questions remain about how telediagnostic RUQ ultrasound fits into the larger healthcare system. This approach to improving access to diagnostic imaging has the potential to optimize clinical decision-making by minimizing over-referral for care or treatment (when there is not a problem) and reducing referral delays when there is a problem requiring urgent or emergent action. It could improve the accuracy and timeliness of treatment initiation, given that for some individuals the cost (in time and money) of seeking care at all or at higher level facilities may be prohibitive, so a problem may be left unaddressed until it worsens, leading to greater disability and cost. Additionally, there may be greater trust in locally based diagnostic tools deployed by individuals known in the community, reducing a barrier to care-seeking in some communities. Healthcare delivery worldwide is diverse and different communities have different needs and considerations. Effective deployment will require careful consideration as to the specific population characteristics and health infrastructure of each community. From a medical-legal perspective, patients and providers would need to be informed of the advantages and shortcomings of this approach when using RUQ ultrasound telediagnosis. Similar precedents already exist for the use of point of care ultrasound in emergency settings, and previous studies have not identified litigation related to its use [21, 22].

The enthusiasm for the potential in these results should be tempered with an honest discussion of the limitations. In this study, approximately 1/3 of the examinations were rated by the diagnostic radiologists as “poor” image quality. While the limitations on image quality should certainly be addressed in future studies, they should also be put into context. In clinical practice, a “poor” image quality US exam can be repeated without harm to the patient, and in many cases even the “poor” image quality exams provided our radiologists with some diagnostic information. For example, among the 4 patients with gallstones who had examinations rated of “poor” image quality, the gallstones were still detected in two of the exams, illustrating that even “poor” imaging quality still can afford diagnostic value. Finally, sensitivity and specificity for cholelithiasis were also high even when considering the examinations of “poor” quality.

The “poor” image quality exams were scattered throughout the data collection period, and analysis showed no significant change in image quality over the course of the study. Patient factors such as body habitus clearly affect ultrasound image quality in general, but patient weight or BMI was not recorded in our sample to specifically analyze its impact, which is a limitation of our study. BMI is a well-known determinant of ultrasound image quality and this will be important to consider in future studies of quality [23]. Failure of the patient to take a deep inspiration during sweeps and failure to perform the VSI with proper technique likely also explain some of the “poor”-quality exams. The relatively low image quality of the DP-10 used in the study was likely another cause of “poor”-quality exams. Like any ultrasound exam, VSI is operator-dependent and more thorough training may assist in producing more reliable results. There was no feedback provided to the operators during the course of the study to address image quality. Had a quality improvement program been in place, re-training the operator could have occurred after a sub-optimal exam. In the future, artificial intelligence may be able to assist in providing real-time feedback to the operator.

The radiologists interpreting the VSI found that the majority of “poor”-quality exams did not visualize the superior aspect of the liver. To address this limitation, a potential modification to the protocol with sweeps that start at the nipple rather than the costal margin could be performed. Although this would ensure the superior liver was included in the field of view, an anticipated drawback would be rib shadowing. It is possible that sweeps starting from the nipple could be added in conjunction with the existing protocol sweeps for greater redundancy. An extra 1–2 sagittal sweeps would also likely improve redundancy and add value to the examination (at the expense of a slightly longer exam time).

In future studies, it would also be helpful to study a population presenting with gastrointestinal symptoms. The number of abnormal cases in our sample was small, leading to statistical uncertainty about concordance, especially for abnormalities aside from gallstones. As a further limitation, only the report of a single radiologist constituted “ground truth.” Few images were captured by the radiologist during the gold standard examination, making it difficult to verify his findings when the radiology report and VSI interpretation were discordant. Future studies including a more rigorous reference standard like computed tomography might be considered. Nonetheless, considering agreement was quite high between VSI and standard of care at baseline, this likely did not significantly impact our conclusions. Given that the sonographic appearance of the RUQ is similar for males and females, our female majority sample would not be expected to affect the overall conclusions, but future studies with more equal representation among males and females would be ideal. Especially given the primary goal of our study was to demonstrate feasibility of RUQ teleultrasound, it should be mentioned that these limitations are thought to have minimal impact on our conclusions regarding image quality and organ visualization.

## Conclusions

For the modest price of a tablet and portable ultrasound machine, telediagnostic RUQ ultrasound can be delivered to a low-resource community and deployed after just a few hours of training. This RUQ telediagnostic system offers a promising opportunity to improve the health of the global community. While its exact role in the overall healthcare system remains to be fully elucidated, it is conceivable that RUQ VSI telediagnostic ultrasound may be used as an alternative to the traditional RUQ ultrasound in areas that lack experienced operators to detect hepatic and biliary pathology. Further study should be undertaken with improved versions of the RUQ protocol and with patients experiencing gastrointestinal symptoms. Despite the limitations, the results of this study suggest that our RUQ VSI telediagnostic system offers the ability to diagnose pathology and has the potential to improve access to imaging worldwide.

## Supporting information

S1 Table Telediagnostic system features [15]. (DOCX)

S2 Table Basic demographic and scan information. (DOCX)

S3 Table File size and sweep length in relation to image quality. (DOCX)

S4 Table Image quality and sweep length regression. (DOCX)

S5 Table Confidence versus image quality for VSI. For all imaging sites, reader-reported confidence varied based on image quality. Confidence was scored on a 1–3 scale (1 = low confidence, 2 = moderate confidence, 3 = high confidence), with higher quality images tending to result in greater confidence. Reported values are confidence as median (range). P values are results of chi-square test.
(DOCX)

S6 Table Agreement on all data between VSI and standard of care. Reported values are percentage (95% confidence interval), with Cohen’s kappa (95% confidence interval) for reader agreement. P values are results of comparing kappa to a theoretical mean of 0.
(DOCX)

S1 File RUQ training video. (MOV)

S2 File Normal VSI exam. Sweeps submitted from this case correspond to the negative VSI scan in Fig 3. A-c correspond to the sweeps as seen in Fig 2.
(ZIP)

S3 File VSI sweep of cholelithiasis. Cine clip of the transverse sweep through the cholelithiasis visualized in Fig 4.
(MP4)
