# Supplementary material for: Testing telediagnostic right upper quadrant abdominal ultrasound in Peru: A new horizon in expanding access to imaging in rural and underserved areas
Source: PLoS One. 2021 Aug 11;16(8):e0255919. doi: 10.1371/journal.pone.0255919 (PMC8357175; doi:10.1371/journal.pone.0255919)
Supplement: S1 Table — (DOCX) [file pone.0255919.s001.docx]

| Feature | Significance |
| --- | --- |
| Asynchronicity | Images are acquired without supervision of a specialist and may be interpreted later by a clinician or diagnostic radiologist. Images can be obtained without an active internet connection and sent at a later time when a network is available. Real-time synchronous video systems require higher bandwidth and presence of the interpreting physician during the examination which is generally not feasible in many rural communities. |
| Ease of Use | Users are guided into performing the VSI exams and sending the telemedicine acquisitions with a tablet. Previous telemedicine platforms have sometimes been limited by technical complexity (14, 21). |
| Integration of Image Acquisition, Telemedicine Platform, Interpretation, and Reporting | VSI protocols, telemedicine platform, and specialist interpretation are all integrated in a single system. |
| Low Bandwidth Requirement | Cine clips are compressed and may be sent over even bandwidths as low as dial-up Internet. |
| Low Cost | Tablets and portable ultrasound machines can be feasibly purchased for use in low resource communities. |
| Scalability | Standardization of the scan protocols and training facilitate the capacity for widespread deployment. |
| Short Training | Users can be trained on both image acquisition and the telemedicine software in a few hours. Traditional sonographic training can take years. |
| Volume Sweep Imaging (VSI) Acquisitions | Images may be obtained by individuals with no prior ultrasound training, and protocols are based on external body landmarks. |
