# Supplementary material for: Testing telediagnostic right upper quadrant abdominal ultrasound in Peru: A new horizon in expanding access to imaging in rural and underserved areas
Source: PLoS One. 2021 Aug 11;16(8):e0255919. doi: 10.1371/journal.pone.0255919 (PMC8357175; doi:10.1371/journal.pone.0255919)
Supplement: S2 Table — (DOCX) [file pone.0255919.s002.docx]

Basic Demographic and Scan Information.

|  | Number of Scans (Percentage of Total) | Female | Male | Age |
| --- | --- | --- | --- | --- |
| Total Scans | 144 (100%) | 129 | 15 | 43.9±20.2 |
| Poor Image Quality | 53 (36.8%) | 49 | 4 | 42.7±21.9 |
| Acceptable Image Quality | 56 (38.9%) | 53 | 3 | 43.3±18.8 |
| Excellent Image Quality | 35 (24.3%) | 27 | 8 | 46.6±20.0 |
| Acceptable/Excellent Image Quality | 91 (63.2%) | 80 | 11 | 44.6±19.3 |
