# Supplementary material for: Testing telediagnostic right upper quadrant abdominal ultrasound in Peru: A new horizon in expanding access to imaging in rural and underserved areas
Source: PLoS One. 2021 Aug 11;16(8):e0255919. doi: 10.1371/journal.pone.0255919 (PMC8357175; doi:10.1371/journal.pone.0255919)
Supplement: S3 Table — (DOCX) [file pone.0255919.s003.docx]

File Size and Sweep Length in Relation to Image Quality.

|  | File Size (MB) | Sweep Length 1 (s) | Sweep Length 2 (s) | Sweep Length 3 (s) | Sweep Length 4 (s) | Sweep Length 5 (s) | Sweep Length 6 (s) |
| --- | --- | --- | --- | --- | --- | --- | --- |
| Total Scans | 7.63±1.46 | 9.44±2.83 | 8.83±2.66 | 8.11±2.88 | 9.31±3.27 | 15.8±4.84 | 12±3.27 |
| Poor Image Quality | 7.56±1.49 | 9.96±3.6 | 8.96±3.22 | 8.67±3.91 | 9.02±3.32 | 14.6±4.88* | 11.7±3.26 |
| Acceptable Image Quality | 7.81±1.41 | 9.21±2.2 | 8.72±2.27 | 7.93±2.13 | 9.63±3.34 | 17.3±4.28*,† | 12.1±3.01 |
| Excellent Image Quality | 7.41±1.5 | 9.03±2.41 | 8.79±2.4 | 7.59±1.94 | 9.21±3.17 | 14.9±5† | 12.1±3.78 |
| Acceptable/Excellent Image Quality | 7.66±1.45 | 9.14±2.27 | 8.75±2.3 | 7.8±2.06 | 9.47±3.27 | 16.4±4.68 | 12.1±3.3 |
| P value (ordinary one-way ANOVA) | 0.42 | 0.24 | 0.89 | 0.19 | 0.61 | 0.006 | 0.72 |

*,† Significant pairwise differences (p<0.05).
