# Supplementary material for: Testing telediagnostic right upper quadrant abdominal ultrasound in Peru: A new horizon in expanding access to imaging in rural and underserved areas
Source: PLoS One. 2021 Aug 11;16(8):e0255919. doi: 10.1371/journal.pone.0255919 (PMC8357175; doi:10.1371/journal.pone.0255919)
Supplement: S4 Table — (DOCX) [file pone.0255919.s004.docx]

Image Quality and Sweep Length Regression.

| Outcome | Predictor | Comparison | Odds Ratio (95% confidence interval) | P value |
| --- | --- | --- | --- | --- |
| Image Quality | Date of Scan | Excellent vs. Poor | 1.00 (0.99-1.01) | 0.91 |
|  |  | Acceptable vs. Poor | 1.01 (1.00-1.01) | 0.045 |
|  |  | Acceptable/Excellent vs. Poor | 1.00 (0.99-1.01) | 0.18 |
|  | Time of Scan | Excellent vs. Poor | 0.79 (0.42-1.49) | 0.79 |
|  |  | Acceptable vs. Poor | 1.15 (0.67-1.99) | 0.62 |
|  |  | Acceptable/Excellent vs. Poor | 0.99 (0.61-1.64) | 0.99 |
| Outcome | Predictor | Sweep Length | B (95% confidence interval) | P value |
| Sweep Length | Date of Scan | 1 | -0.003 (-0.009-0.002) | 0.25 |
|  |  | 2 | -0.001 (-0.006-0.004) | 0.68 |
|  |  | 3 | -0.002 (-0.008-0.004) | 0.52 |
|  |  | 4 | 0.002 (-0.005-0.008) | 0.59 |
|  |  | 5 | 0.005 (-0.005-0.014) | 0.35 |
|  |  | 6 | -0.003 (-0.009-0.004) | 0.40 |
|  | Time of Scan | 1 | -0.023 (-0.69-0.65) | 0.95 |
|  |  | 2 | -0.10 (-0.73-0.52) | 0.75 |
|  |  | 3 | 0.035 (-0.65-0.72) | 0.92 |
|  |  | 4 | 0.066 (-0.71-0.84) | 0.87 |
|  |  | 5 | 0.16 (-0.98-1.30) | 0.78 |
|  |  | 6 | -0.24 (-1.01-0.54) | 0.55 |
