# Supplementary material for: Testing telediagnostic right upper quadrant abdominal ultrasound in Peru: A new horizon in expanding access to imaging in rural and underserved areas
Source: PLoS One. 2021 Aug 11;16(8):e0255919. doi: 10.1371/journal.pone.0255919 (PMC8357175; doi:10.1371/journal.pone.0255919)
Supplement: S5 Table — For all imaging sites, reader-reported confidence varied based on image quality. Confidence was scored on a 1–3 scale (1 = low confidence, 2 = moderate confidence, 3 = high confidence), with higher quality images tending to result in greater confidence. Reported values are confidence as median (range). P values are results of chi-square test. (DOCX) [file pone.0255919.s005.docx]

**Confidence versus Image Quality for VSI.** For all imaging sites, reader-reported confidence varied based on image quality. Confidence was scored on a 1-3 scale (1= low confidence, 2= moderate confidence, 3= high confidence), with higher quality images tending to result in greater confidence. Reported values are confidence as median (range). P values are results of chi-square test.

| Measure | Poor Image Quality (n=53) | Acceptable Image Quality (n=56) | Excellent Image Quality (n=35) | P value |
| --- | --- | --- | --- | --- |
| Liver | 1(1-3) | 3(1-3) | 3(2-3) | <0.0001 |
| Gallbladder | 1(1-3) | 3(1-3) | 3(1-3) | <0.0001 |
| Pancreas | 1(1-3) | 1(1-3) | 3(1-3) | <0.0001 |
| Right Kidney | 1(1-3) | 2(1-3) | 3(2-3) | <0.0001 |
| Large Vessels | 1(1-3) | 2(1-3) | 3(1-3) | <0.0001 |
| Exam | 1(1-3) | 2(1-3) | 3(2-3) | <0.0001 |
