# Supplementary material for: Testing telediagnostic right upper quadrant abdominal ultrasound in Peru: A new horizon in expanding access to imaging in rural and underserved areas
Source: PLoS One. 2021 Aug 11;16(8):e0255919. doi: 10.1371/journal.pone.0255919 (PMC8357175; doi:10.1371/journal.pone.0255919)
Supplement: S6 Table — Reported values are percentage (95% confidence interval), with Cohen’s kappa (95% confidence interval) for reader agreement. P values are results of comparing kappa to a theoretical mean of 0. (DOCX) [file pone.0255919.s006.docx]

**Agreement on All Data Between VSI and Standard of Care.** Reported values are percentage (95% confidence interval), with Cohen’s kappa (95% confidence interval) for reader agreement. P values are results of comparing kappa to a theoretical mean of 0.

| Measure | VSI | Standard of Care | Overall Agreement | Overall agreement (ignoring non-visualized cases) | Cohen’s kappa (95% confidence interval) | Cohen’s kappa (ignoring non-visualized cases) | P value | P value (ignoring non-visualized cases) |
| --- | --- | --- | --- | --- | --- | --- | --- | --- |
| Liver Echogenicity (% Normal) | 84.7% (77.8-90.2%) | 82.6% (75.4-88.4%) | 99.3% | 99.3% | 0.92(0.84-1) | 0.92(0.84-1) | <0.0001 | <0.0001 |
| Liver Abnormal | 1.39% (0.169-4.93%) | 2.08% (0.432-5.97%) | 86.1% | 99.2% | 0.15(-0.042-0.34) | 0.8(0.41-1.2) | <0.0001 | <0.0001 |
| Gallbladder Abnormal | 13.9% (8.69-20.6%) | 14.6% (9.26-21.4%) | 70.1% | 92.7% | 0.45(0.35-0.55) | 0.77(0.62-0.92) | <0.0001 | <0.0001 |
| Pancreas Abnormal | 0% (0-2.53%) | 0% (0-2.53%) | 43.4% | 100% | 0* | 1* | 1* | <0.0001* |
| Right Kidney Abnormal | 1.39% (0.169-4.93%) | 1.39% (0.169-4.93%) | 65.2% | 98.9% | 0.046(-0.021-0.11) | 0.66(0.033-1.3) | 0.002 | <0.0001 |
| Exam Abnormal | 15.3% (9.83-22.2%) | 17.4% (11.6-24.6%) | 94.0% | 94.0% | 0.79(0.65-0.93) | 0.79(0.65-0.93) | <0.0001 | <0.0001 |
